# Flies evolved small bodies and cells at high or fluctuating temperatures

**DOI:** 10.1002/ece3.2534

**Published:** 2016-10-12

**Authors:** Gregory J. Adrian, Marcin Czarnoleski, Michael J. Angilletta

**Affiliations:** ^1^ School of Life SciencesArizona State UniversityTempeAZUSA; ^2^ Institute of Environmental SciencesJagiellonian UniversityKrakowPoland

**Keywords:** body size, cell size, *Drosophila*, experimental evolution, fluctuations, selection, temperature

## Abstract

Recent theory predicts that the sizes of cells will evolve according to fluctuations in body temperature. Smaller cells speed metabolism during periods of warming but require more energy to maintain and repair. To evaluate this theory, we studied the evolution of cell size in populations of *Drosophila melanogaster* held at either a constant temperature (16°C or 25°C) or fluctuating temperatures (16 and 25°C). Populations that evolved at fluctuating temperatures or a constant 25°C developed smaller thoraxes, wings, and cells than did flies exposed to a constant 16°C. The cells of flies from fluctuating environments were intermediate in size to those of flies from constant environments. Most genetic variation in cell size was independent of variation in wing size, suggesting that cell size was a target of selection. These evolutionary patterns accord with patterns of developmental plasticity documented previously. Future studies should focus on the mechanisms that underlie the selective advantage of small cells at high or fluctuating temperatures.

## 1 Introduction

Cells are a key feature of life, but they differ dramatically in structure and function within and among organisms. Much of this variation has obvious biological significance, but we still do not understand the selective pressures that influence properties such as cell volume. Arguably, variation in cell volume has been understudied as a potential driver of physiological performance. A provocative theory of metabolism even makes a bold and erroneous assumption that cells are of the same size in all organisms (West & Brown, 2005). Quite the opposite, cell size has varied among tissues, individuals, or species whenever biologists have focused their lenses on this subject (Arendt, 2007; Czarnoleski, Cooper, Kierat, & Angilletta, 2013; Kozłowski, Czarnołęski, François‐Krassowska, Maciak, & Pis, 2010; Stevenson, Hill, & Bryant, 1995). The variation in cell size within and among individuals of the same species poses a challenge for evolutionary biologists to explain.

An emerging theory holds that cell size evolves according to a trade‐off between the capacity for and the efficiency of metabolism (Atkinson, Morley, & Hughes, 2006; Czarnoleski, Dragosz‐Kluska, & Angilletta, 2015; Czarnoleski et al., 2013; Kozłowski, Konarzewski, & Gawelczyk, 2003; Szarski, 1983). The optimal size balances the benefit of acquiring resources quickly against the cost of keeping membranes operational. On one hand, a volume of tissue that consists of small cells enjoys a larger area of cell membranes, which enables substrates, products, and signals to diffuse rapidly between cells. A fine network of membranes resulting from small cells should also enable oxygen to permeate tissues more quickly, because oxygen diffuses faster through lipids than through water (Subczynski et al., 1989). Moreover, a greater density of cells provides more nuclei for transcription and shorter distances between the nucleus and other organelles. These metabolic advantages should favor organisms with smaller cells, especially when the capacity for metabolism becomes a limiting factor. On the other hand, the energy required to maintain and repair membranes increases as the area of membranes expands. For example, the larger area of membranes created by small cells requires more energy to defend electrochemical gradients against the diffusion of ions. Smaller cells would also require more energy to remodel when changes in body temperature alter the fluidity. These costs should select against organisms with small cells in environments where supplies of metabolites exceed the demands of cells.

Czarnoleski et al. (2013, 2015) used this theory to predict the optimal cell size at different temperatures. At a low temperature, where metabolism proceeds slowly, large cells should provide a sufficient area of membranes to transport the metabolites needed to sustain life. As the mean or the variance of temperature increases, smaller cells would help to meet the increased metabolic demands of tissues, especially for oxygen and transcripts. These researchers found that flies (*Drosophila melanogaster*) raised at a greater mean or variance of temperature developed smaller cells (Czarnoleski et al., 2013). This plastic response to mean temperature mirrored that seen in other studies of flies (reviewed by Partridge & French, 1996; Angilletta, Steury, & Sears, 2004). However, a plastic response to the variance of temperature had never been studied previously.

Studies of evolutionary process can determine whether the plasticity of cell size reflects adaptation to thermal environments. For example, Partridge et al., (1994) used experimental evolution to show that larger cells evolved in populations of *D. melanogaster* evolving at a lower temperature. Thus, evolution in a cold environment pushed cell size in the same direction as did development in a cold environment. This consistency between developmental and genetic effects bolsters the view that plasticity of cell size confers a selective advantage. However, biologists have no data on the evolution of cell size at fluctuating temperatures. If plastic responses to thermal fluctuations are adaptive, populations evolving experimentally should diverge in mean cell size according to the variance of temperature.

Here, we studied the cell and body sizes of flies from populations that evolved experimentally at either constant or fluctuating temperatures. These flies exhibit genetic variation in morphological (Yeaman, Chen, & Whitlock, 2010), biochemical (Cooper et al., 2010), and physiological traits (Condon et al., 2014; Condon et al., 2015). We estimated the genetic variation in body size and cell size among populations from different selective treatments. Based on previous studies of developmental plasticity, we expected two patterns of variation in cell size among genotypes from different thermal treatments. First, we expected genotypes from a high constant temperature or fluctuating temperatures to develop smaller cells compared to genotypes from a low constant temperature. Second, we expected genotypes from fluctuating temperatures to develop cells intermediate in size to the cells of genotypes from constant temperatures. This second prediction stemmed from the hypothesis that flies at fluctuating temperatures must balance the benefit of small cells during warm periods with the cost of small cells during changes in temperature.

## 2 Methods

We studied experimental populations of *Drosophila melanogaster* created by Yeaman et al. (2010). Their selection experiment comprised five populations evolving at each of three conditions: a constant 16°C, a constant 25°C, or fluctuations between 16 and 25°C among generations. Each population comprised 2,000–4,000 flies in a cage made of nylon mesh (22 cm × 25 cm × 32 cm). Every 2 or 4 weeks at 16°C or 25°C, respectively, a new generation was started by adding fresh bottles of medium to each cage. Temporal fluctuations in temperature were generated by moving cages between 16 and 25°C, or vice versa, at the start of each generation. The photoperiod was 12L: 12D for all populations. Populations were sampled in 2009, when those at 16 and 25°C had completed 32 and 64 generations, respectively; an intermediate number had occurred in populations exposed to fluctuating temperatures. To preserve the genetic diversity within and among populations for future studies, isofemale lines were founded by pairing virgin flies from each population in August of 2009. See previous articles for additional information about the selection experiment (Yeaman et al., 2010) and the isofemale lines (Condon et al., 2014).

Our experiment included an average of six isofemale lines (range = 3–8 lines) from each of the 15 experimental populations. In 2014, we controlled the density of each isofemale line for two generations by transferring only two adults of each sex into new vials to lay for 48 h. Following this period, pairs of 7‐day‐old females from each isofemale line were transferred to fresh vials. These vials were kept at 20.5°C, which is intermediate to the temperatures experienced during experimental evolution. After 48 h, females were removed to limit the density of offspring in each vial. The vials were kept at 20.5°C until offspring emerged as adults. These adults were used in our study of morphology. During the experiment, isofemale lines were maintained in 25 × 90 mm vials containing about 3 cm of standard medium (Bloomington Stock Center, Bloomington, IN, USA).

We measured and photographed flies under a stereomicroscope (Zeiss Stemi 2000‐C). Each fly was placed on a porous pad receiving a continuous flow of carbon dioxide. Flies were manipulated to expose the left shoulder and wing. Surgical scissors were used to remove the wing at its base, as close to its connection with the thorax as possible. The naked thorax was measured from the rear end to the bristle closest to the head. The wing was mounted on a slide with a drop of xylene and a drop of Permount medium.

**Figure 1:**
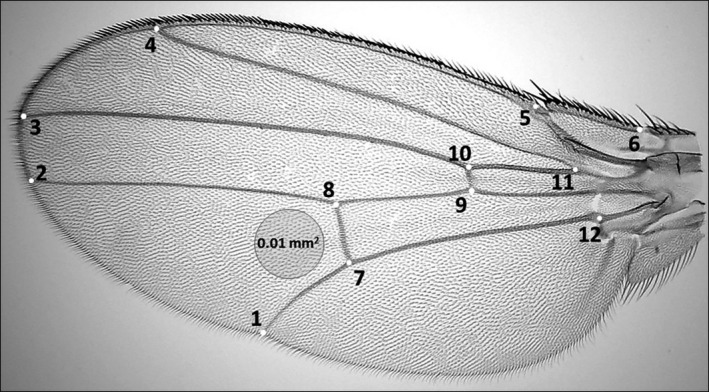
Landmarks on each wing were used to estimate wing size. The mean area of cells was estimated from the density of trichomes in a circular section of the wing (0.01 mm^2^).

We measured the area of each wing and its cells from a digital image. A camera, controlled by computer software (ZEN 2011), enabled us to capture images from a stereomicroscope at 16‐fold magnification. For each wing, we labeled 12 landmarks (Figure 1) used to estimate wing size by the software program tpsDig2 (Rohlf, 2004). This program generates a centroid size for the wing, defined as the square root of the sum of squared coordinates of the landmarks (Hoffmann & Shirriffs, 2002). To calculate the mean cell size for each wing, we counted trichomes in a circle of 0.01 mm^2^ on the distal section of the wing between veins IV and V (Figure 1). The reciprocal of trichome density was then calculated to provide an estimate of the mean area of an epidermal cell (Dobzhansky, 1929).

To quantify sources of variation in the sizes of thoraxes, wings, and cells, we fit general linear mixed models using the nlme library of the R Statistical package (R Development Core Team 2011). Both sex and the selective environment were fixed factors in all analyses. Thorax length was a covariate in the analysis of wing size, and wing size was a covariate in the analysis of cell size. Isofemale line was a random factor, nested within the random factor of experimental population. Parameters were estimated according to Zuur et al., (2009). Following Burnham and Anderson (2002), we used multimodel averaging to estimate the most likely values of means. First, we used the *MuMIn* library (Bartoń, 2013) to fit all possible models to the data. Then, we calculated the Akaike information criterion and Akaike weight of each model, the latter variable being the probability that the model best describes the data. Finally, we calculated the weighted average of each parameter including estimates from all models. The resulting values of parameters were used to calculate the most likely mean for each group. This approach eliminates the need to interpret *p* values, because all models (including the null model) contributed to the most likely value of each mean.

## 3 Results and Discussion

**Figure 2:**
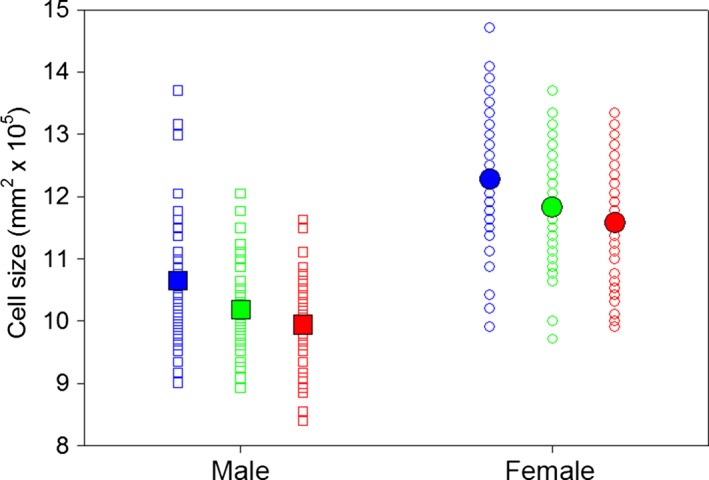
Flies that developed in cold constant (blue symbols) environments developed larger thorax sizes and larger wings than flies raised in both warm constant environments (red symbols) and fluctuating environments (green symbols). For thorax size, females (circles) were more impacted than were males (squares) across the thermal treatments. Large, solid symbols denote the means estimated by multimodel averaging

The mean size of cells diverged among populations of flies evolving at different temperatures. Cells of flies whose ancestors evolved at either 25°C or at fluctuating temperatures were 4%‐6% smaller than cells of flies whose ancestors evolved at 16°C (Figure 2). Because cell size depends on the body size of a fly (Stevenson et al., 1995), natural selection could have enhanced cell size directly or indirectly if cold environments favored larger flies. In the latter case, a genetic correlation between traits implies that cell size would diverge among populations whenever natural selection causes wing size to diverge. At the same time, natural selection of cell size could amplify the rate of evolutionary divergence. Our results indicate that both processes reduced cell size in warm or fluctuating environments. Two of the three most likely models of cell size included wing size as a covariate (Table 1), meaning that some divergence in cell size could have resulted by indirect selection. However, variation in wing size accounts for a tiny fraction of the variation in cell size among selective treatments (Table 2). Thus, cell size diverged primarily from direct selective pressures caused by environmental temperatures. Indeed, previous studies have reported the evolution of small wing cells in flies from populations that had evolved at high temperatures, although these studies involved unreplicated populations (Cavicchi et al., 1985; Noach et al., 1997). Our study, which included five populations per selective treatment, rules out genetic drift as a likely explanation for the evolution of small cells at high temperatures.

**Table 1:** All likely models included an effect of selective treatment on cell size

Terms in the Model	Parameters	Log likelihood	AIC_c_	ΔAIC_c_	Akaike weight
1) Sex + treatment	7	−439.8	893.9	0	0.258
2) Sex + treatment + wing area + (treatment·wing area)	10	−436.8	894.1	0.24	0.229
3) Sex + treatment + wing area	8	−439.5	895.4	1.55	0.119
4) Sex + treatment + (sex·treatment)	9	−438.5	895.6	1.70	0.110
5) Sex + treatment + wing area + (sex·wing area) + (treatment·wing area)	11	−436.7	896.2	2.27	0.083
6) Sex + treatment + wing area + (sex·treatment)	10	−438.4	897.3	3.42	0.047
7) Sex + treatment + wing area + (sex·wing area)	9	−439.5	897.5	3.65	0.042
8) Sex + treatment + wing area + (sex·treatment) + (treatment·wing area)	12	−436.5	897.8	3.91	0.036
9) Sex	5	−444.2	898.5	4.61	0.026

Likely models are ranked according to their Akaike information criterion (*AIC*_*c*_). For each model, we provide the Akaike weight, which equals the probability that the model describes the data better than the other models. All models contained an intercept and error terms associated with population and isofemale line.

**Table 2:** Means of traits for each group of flies estimated from multimodel averaging

Trait	Males			Females		
	16°C	25°C	16/25°C	16°C	25°C	16/25°C
Thorax length	6.90	6.54	6.68	12.22	10.83	11.12
Wing centroid	1775	1756	1734	2008	1962	1958
Cell area	10.56	9.95	10.23	12.17	11.54	11.80
Cell area (adjusted for wing centroid)	10.60	9.95	10.22	12.29	11.58	11.78

Flies were from populations that had evolved at a constant 16°C, a constant 25°C, or fluctuations between these temperatures (16/25°C). To assess direct selection on cell size, we also report cell areas adjusted for variation in wing size. This adjustment had little effect on the mean cell size of each group, suggesting that direct selection was stronger than indirect selection.

Generally, one can interpret a divergence in cell size among constant and fluctuating environments in two ways. On one hand, populations in the fluctuating treatment experience a greater thermal variance, which can select for a certain cell size. On the other hand, these populations also experience a mean temperature in between the temperatures of the constant treatments, which can select for an intermediate cell size. For our experiment, we favor the former interpretation because flies in our fluctuating treatment experienced either 16°C or 25°C, but never experienced the mean temperature of 20.5°C. Thus, at no point in time could natural selection have favored genotypes that perform well at 20.5°C. Instead, populations could have adapted to being held at 25°C in some generations and at 16°C in other generations (hence, to the variance of temperature), as alleles conferring greater fitness under such conditions became more frequent. Interpreted in this way, our results support the view that either high or fluctuating temperatures favor smaller cells, which facilitate metabolic activity through a greater surface area of membranes. At fluctuating temperatures, generations at 25°C experienced greater demand for metabolic substrates than did generations at 16°C. However, any selective pressure created by this demand would have been offset by the energetic cost of membranes in the other generations. Thus, generations at 16°C, which faced low metabolic demands, probably fared better with larger cells than did generations at 25°C. If we surmised these selective pressures correctly, the intermediate cell size of genotypes from the fluctuating treatment reflects a compromise between performance at 25°C and efficiency at 16°C.

**Figure 3:**
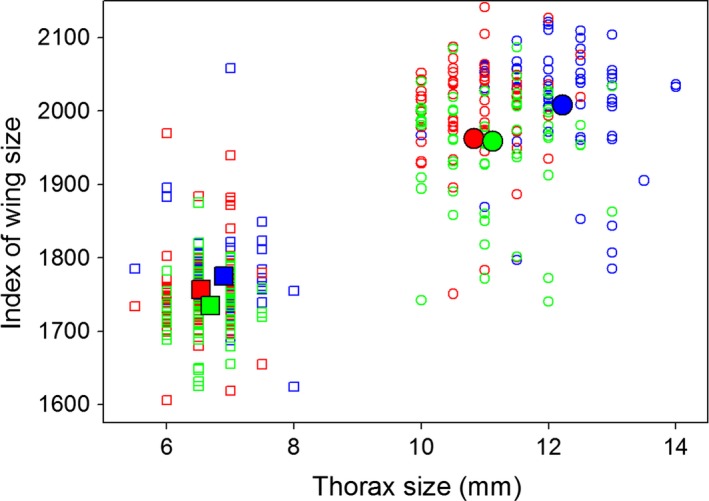
Flies that developed in a constant cold environment (blue symbols) had larger cells than flies developing in constant hot environments (red symbols). Flies that developed in fluctuating environments (green symbols) had cells intermediate in size. Large, solid symbols denote the means estimated by multimodel averaging

Mean wing size also diverged among populations, but not exactly as predicted (Figure 3). Consistent with our hypothesis, mean wing size was largest in flies from 16°C. However, the smallest mean wing size was observed in flies from the fluctuating environment rather than flies from the warm environment. Flies from the fluctuating environment had wings averaging 2%–3% smaller than did flies from the cold environment. This pattern emerged despite an inability to detect variation in wing size among these populations in previous experiments (Condon et al., 2014; Yeaman et al., 2010); the discrepancy could have resulted from sampling error or a difference in developmental temperatures used in our study (20.5°C) and previous studies (16 and 25°C). If the pattern that we found holds, thermal variation might affect the evolution of wing size independently of the mean temperature. If so, this evolutionary response would match the developmental response reported by Czarnoleski et al. (2013, 2015). These researchers raised flies at either constant or fluctuating temperatures, while controlling the mean temperature. Flies from the fluctuating environments had smaller wings than those from the constant environments. This result was probably due to disproportional effects of high and low temperatures on development, rather than thermal fluctuations per se. When Czarnoleski et al., (2015) raised flies at temperatures that fluctuated either infrequently or frequently, while controlling the mean and variance of temperature, they saw no significant effect of thermal fluctuations on thorax or wing size. Therefore, thermal variation must act primarily by increasing exposure to high temperatures, which cause flies to develop smaller wings.

The patterns of cell size among our treatments imply that *Drosophila melanogaster* evolved larger wings through larger cells, rather than more cells. This observation agrees with that of Partridge et al., (1999), who also concluded that larger wings evolved primarily through larger cells in a selection experiment with two constant thermal treatments. These researchers considered their result unusual, because larger flies usually result from more cells. However, comparative studies have since shown that either the number or size of cells can underlie genetic variation in body size (reviewed by Angilletta et al., 2004). What matters is whether body size or cell size responds to selection when the two can evolve independently. Our experiment, in which cell size appears to evolve primarily through direct selection, supports the hypothesis that large cells represent an adaptation to low temperature, rather than a means of making a large wing. Nevertheless, a larger thorax and a larger wing were clearly favored in the cold environment relative to the warm or the fluctuating environment. These observations agree with those of other researchers, who reported that low temperatures cause flies to develop larger legs, eyes, or wings through the growth of epidermal cells (Azevedo et al., 2002; Czarnoleski et al., 2013).

Consistent with previous observations (Anderson, 1966; Partridge et al., 1994), thorax size diverged genetically between populations at 25°C and populations at 16°C. After both sets of flies developed at an intermediate temperature, flies derived from the warmer selective environment had smaller thoraxes on average (Figure 3). Females exhibited a more pronounced difference in mean thorax length than males did, with females and males from the warmer selection lines being 11% and 5% smaller, respectively, than those from the cold lines. More interestingly, the mean thorax size of flies derived from the fluctuating selective environment lay in between that of flies derived from the warm and cold selective environments, which means that the variation in mean thorax size among treatments matched the predicted ranking: 16°C lines >16/25°C lines >25°C lines. For females, the mean thorax length of flies from the fluctuating environment was 9% smaller than that of flies from the cold environment and 3% larger than that of flies from the warm environment (Figure 3).

The evolutionary divergence in body size reinforces the view that colder environments favor delayed maturation at a larger size. Comparative analyses of phenotypic plasticity in laboratory experiments (Atkinson, 1994) and genetic variation along latitudinal clines (Huey et al., 2000) established that most ectotherms mature at larger sizes in colder environments. This phenotypic plasticity has been considered a product of physical constraints as well as natural selection (Angilletta et al., 2004; Atkinson & Sibly, 1997). Explanations based on physical constraints have focused on whether and why cells must be smaller at high temperatures (Berrigan & Charnov, 1994; vanVoorhies, 1996). By contrast, explanations based on natural selection have focused on whether warm environments impose a greater risk of mortality (Angilletta et al., 2004; Kozłowski et al., 2004), enhance the relationship between body size and fecundity (Arendt, 2011), or change size‐dependence of somatic production (Kozłowski et al., 2004). The repeated evolution of body size among several selection experiments bolsters the view that natural selection causes a larger size at maturity to evolve in colder environments, at least in *Drosophila melanogaster* (Angilletta et al., 2004; Atkinson & Sibly, 1997; Partridge & French, 1996).

Although selection experiments offer substantial advantages over comparative analyses of natural populations, we still do not know whether the patterns emerging from these experiments can be generalized across genetic and environmental backgrounds (Kawecki et al., 2012). The amount of genetic variation, physical linkage of alleles, and epistatic interactions likely vary among populations sampled for selection experiments. Outcomes can also depend on interactions between selective factors of interest, such as temperature, and those that are controlled within experiments but not controlled among them (e.g., nutrition, humidity, density). Given these sources of variation, the predictable genetic divergence in body and cell sizes between our cold and warm populations confirms that environmental temperature is a major factor generating latitudinal clines in body size and cell size of *Drosophila melanogaster* (Zwaan et al., 2000; reviewed by Arendt, 2007). Additionally, we now know that flies at fluctuating temperatures evolve thorax and cell sizes similar to those of flies at intermediate temperatures. These patterns of adaptation underscore the need to build a comprehensive theory of optimal cell size. Such a theory should incorporate mechanisms that can explain the evolution of small bodies and cells in warm or variable environments, as well as the thermal plasticity of these phenotypes.

## Data archival

Authors will deposit data to Dryad.
